# Ninth Annual Meeting of the Tri-State Medical Society

**Published:** 1897-10

**Authors:** 


### NINTH ANNUAL MEETING OF THE TRI-STATE MEDICAL SOCIETY.

The ninth annual meeting of the Tri-State Medical Society of Alabama, Georgia, and Tennessee, will be held at The Tulane, Nashville, Tenn., Tuesday, Wednesday, and Thursday, Oct., 12, 13 and 14, 1897.

OFFICERS:

President—W. F. WESTMORELAND . . . . . Atlanta, Ga.
Vice-Presidents—M. B. HUTCHINS . . . . . Atlanta, Ga.
SEALE HARRIS . . . . Union Springs, Ala.
C. R. ACHISON . . . . . Nashville, Tenn.
Secretary—FRANK TRESTER SMITH . 826 Market St., Chattanooga, Tenn.
Treasurer—GEO. R. WEST . . . . . . . Chattanooga, Tenn.
Chairman Committee of Arrangements—W. D. HAGGARD, Jr., Nashville, Tenn.

PROGRAM.

TUESDAY, OCTOBER 12, 1897—MORNING.

Registration, Introductions, etc., 9:00 to 10:00 A. M.
Prayer—Rev. J. B. Hawthorne.
Address of Welcome—Gov. R. L. Taylor.—Response by F. M. Ridley, M. D.
Reading of Papers and Discussions, 10:00 to 12:00 A. M.

AFTERNOON, 2:00 TO 5:00.

Reading of Papers and Discussions.

PROGRAM—Continued.

NIGHT, 7:45.

Concert by Conterno's Band and Fireworks at Centennial Grounds.

WEDNESDAY, OCTOBER 13—MORNING, 9:00 TO 12:00.

Reading of Papers and Discussions.

AFTERNOON.

Tri-State Medical Society Day at Tennessee Centennial.

NIGHT, 7:30 TO 9:00.

Reading of Papers and Discussions.

Exhibition of X-Ray Apparatus.

THURSDAY, OCTOBER 14—MORNING, 9:00 TO 12:00.

Reading of Papers and Discussions.

Election of Officers.

AFTERNOON, 2:00 TO 5:00.

Reading of Papers and Discussions.

NIGHT, 7:30 TO 9:00.

Reading of Papers and Discussions.

LIST OF PAPERS.

1. President's Address: "Carcinoma of the Breast." W. F. Westmoreland, Atlanta, Ga.
2. "Psychology." J. B. Cowan, Tullahoma, Tenn.
3. "The True Physician—His Responsibilities—His Duty to his Profession, and the People." John C. Legrand, Anniston, Ala.
4. "A Pessimistic and an Optimistic View of Medicine." Y. L. Abernathy, Hill City, Tenn.
5. "A Boq et of Remedial Agencies." John P. Stewart, Attalla, Ala.
6. "Treatment of Typhoid Fever." Jno. A. Larrabee, Louisville, Ky.
7. "Abortive Treatment of La Grippe." E H. Richardson, Atlanta, Ga.
8. "Electro-Therapy in Medicines." Louise Eleanor Smith, Chattanooga.
9. "A New Mode of Internal Electro-Therapy." R P. Johnson, Chattanooga, Tenn.
10. "Common Sore Throat." James H. Atlee, Chattanooga, Tenn.
11. "Vaccination." Seale Harris, Union Springs, Ala.
12. "The Pathology and Diagnosis of Early Phthisis." Llewellyn P. Barbour, Tullahoma, Tenn.
13. "Sero-Therapy in Tuberculosis." Paul Paquin, St. Louis.
14. "Importance of Early Recognition of Pleural Effusions, Due to Causes Other than Those Located in the Pleurae." Louis H. Jones, Atlanta, Georgia.
15. "Abnormal Metabolism." G. W. Drake, Chattanooga, Tenn.
16. "The Subject of Haematology." E. C. Anderson, Chattanooga, Tenn.
17. "Inhibition, Physiological and Pathological." J. F. Peavy, Atmore, Ala.
18. "The Rational Treatment of Cancer of the Uterus." Geo. Wiley Broome, St. Louis, Mo.
19. "A Case of Fracture of the Skull, Followed by Basilar Hemorrhage; Trephining; Recovery." Curran Pope, Louisville, Ky.
20. "The Relation of the Cause to the Immediate and Remote Results of Fracture." R. M. Cunningham, Birmingham, Ala.
21. "Is Cancer Contagious?" E. Mather, Patterson, N. J.

22. "Stimulants and Narcotics in Obstetrics and Gynecology." R. R. Kime, Atlanta, Ga.
23. "Surgical Shock." Gilbert I. Cullen, Cincinnati, O.
24. "Cystitis; Its Course and Treatment." W. L. Nolen, Chattanooga, Tenn.
25. "Stricture of the Urethra and its Treatment." Wm. R. Blue, Louisville, Ky.
26. "Operative Treatment in Enlarged Prostate." H. H. Grant, Louisville, Ky.
27. "Treatment of Chancroidal Ulcers." A. R. Danforth, Atlanta, Ga.
28. "Ablation of the Scrotum for Conditions other than Varicocele." W. S. Goldsmith, Atlanta, Ga.
29. "The Treatment of Cancer of the Rectum." J. M. Mathews, Louisville, Ky.
30. "Burns and Scalds." G. A. Baxter, Chattanooga, Tenn.
31. "Metatarsalgia, or Morton's Painful Toe." Geo. S. Brown, Birmingham, Ala.
32. "The Application of Plaster Jacket and Dressings." F. B. Sloan, Cowan, Tenn.
33. "Surgery of the Stomach." H. Berlin, Chattanooga, Tenn.
34. "Report of Operative Cases (Brain)." S. G. Courtney Pinckney, Atlanta, Ga.
25. "Fracture of the Elbow." B. G. Copeland, Birmingham, Ala.
36. "Treatment of Fractured Maxillaries." D. S. Arnold, Atlanta, Ga.
37. "The After Treatment of Abdominal Operations." Valentine Taliaferro, Atlanta, Ga,
38. "Epiplocele, Report of a Case." J. W. Griggs, West Point, Ga.
39. "Appendicitis." G. Manning Ellis, Chattanooga, Tenn.
40. "The Evolution of the Treatment of the Stump, in Operations for Appendicitis." W. D. Haggard, Jr., Nashville, Tenn.
41. "Obstetrics." W. G. Bogart, Chattanooga, Tenn.
42. "Some of the Mammoth Ovarian Tumors of Surgical History." A. M. Cartledge, Louisville, Ky.
43. "Cases of Ectopic Gestation, Operated on by the Vaginal Route." W. E. B. Davis, Birmingham, Ala.
44. "The Technique in Hysterectomy for Uterine Myomata." W. H. Wathen, Louisville, Ky.
45. "Hysterectomy." Louis Frank, M. D., Louisville, Ky.
46. "Hysterectomy in the Treatment of Pelvic Diseases." Geo. R. West, Chattanooga, Tenn.
47. "Flap Operation for Atresia Vaginae." Geo. H. Noble, Atlanta, Ga.
48. "Meningitis in Children." J. W. Russey, Chattanooga, Tenn.
49. "Mouth Breathing in Children and its Dangers." Wm. Cheatham, Louisville, Ky.
50. "Injurious Results of Incompetent Refraction Work." Frank Sims, Atlanta, Ga.
51. "The Causes, Diagnosis, and Prognosis of Valvular Diseases." Hazle Padgett, Columbia, Tenn.
52. "Rheumatism as an Etiological Factor in Cardiac Diseases." S. W. Fain, Chattanooga, Tenn.
53. .................. Hunter McGuire, Richmond, Va.
54. .................. J. R. Wells, Stone Mountain, Ga.
55. "The Treatment of General Peritonitis." L. S. McMurtry, Louisville, Ky.
56. Address: "Aslyum Reform from the Gynecic Standpoint." Charles A. L. Reed, Cincinnati, O.
57. .................. Wm. Warren Potter, Buffalo, N. Y.
58. .................. Geo. Ben Johnson, Richmond, Va.
59. "Pot-Hunter in Surgery." Joseph Price, Philadelphia, Pa.
60. .................. Joseph A. White, Richmond, Va.
61. "Reports of Some Cases of Abdominal Surgery." James M. Black, Knoxville, Tenn.

62. "Abscess of Thigh and Pelvic Cavity, with Report of a Case." B. P. Key, Tracy City, Tenn.
63. "How to Elevate the Profession in the Country." T. S. Hughes, Cohutta, Ga.
64. Subject to be announced. A. W. Calhoun, Atlanta, Ga.
65. "Various Treatments of Epithelioma." M. B. Hutchins, Atlanta, Ga.
66. "Posterior Urethritis." R. H. Tatum, Chattanooga, Tenn.
67. "Stricture of the Nasal Duct." B. F. Travis, Chattanooga, Tenn.
68. "Chloroform." Cooper Holtzclaw, Chattanooga, Tenn.
69. "Cataract Operations." James Moores Ball, St. Louis, Mo.
70. "An Original Method of Preparing Catgut for Ligatures and Sutures." St. Jos. B. Graham, Savannah, Ga.
71. "Leukaemia and Report of a Case of Hodgkin's Disease." A. W. Mardis, Lebanon, O.
72. "Quinine; Its Use and Abuse in Pregnancy." D. S. Middleton, Rising Fawn, Ga.
73. "Some Remarks of Appendicitis." J. A. Goggins, Alexander City, Ala.
74. "The Practical Application of the Microscope to Every-Day Practice." J. W. Hooper, New Site, Ala.
75. "Formaldehyde as a Disinfectant, with Demonstrations." C. M. Peake, M. D., Atlanta, Ga.
76. Minor D. Mildrell, Chattanooga, Tenn

